# Estimates of total neuron number show that neonatal ethanol causes immediate and lasting neuron loss in cortical and subcortical areas

**DOI:** 10.3389/fnins.2023.1186529

**Published:** 2023-05-02

**Authors:** John F. Smiley, Cynthia Bleiwas, Brandon M. Marino, Prerana Vaddi, Stefanie Canals-Baker, Donald A. Wilson, Mariko Saito

**Affiliations:** ^1^Nathan Kline Institute for Psychiatric Research, Orangeburg, NY, United States; ^2^Department of Psychiatry, School of Medicine, New York University, New York, NY, United States; ^3^Department of Child and Adolescent Psychiatry, School of Medicine, New York University, New York, NY, United States; ^4^Department of Neuroscience and Physiology, School of Medicine, New York University, New York, NY, United States

**Keywords:** fetal alcohol (FAS FASD), apoptosis, thalamus, cerebral cortex, subiculum, medial septum and diagonal band of broca, mammillary body, cingulate cortex

## Abstract

In neonatal brain development there is a period of normal apoptotic cell death that regulates adult neuron number. At approximately the same period, ethanol exposure can cause a dramatic spike in apoptotic cell death. While ethanol-induced apoptosis has been shown to reduce adult neuron number, questions remain about the regional selectivity of the ethanol effect, and whether the brain might have some capacity to overcome the initial neuron loss. The present study used stereological cell counting to compare cumulative neuron loss 8 h after postnatal day 7 (P7) ethanol treatment to that of animals left to mature to adulthood (P70). Across several brain regions we found that the reduction of total neuron number after 8 h was as large as that of adult animals. Comparison between regions revealed that some areas are more vulnerable, with neuron loss in the anterior thalamic nuclei > the medial septum/vertical diagonal band, dorsal subiculum, and dorsal lateral geniculate nucleus > the mammillary bodies and cingulate cortex > whole neocortex. In contrast to estimates of total neuron number, estimates of apoptotic cell number in Nissl-stained sections at 8 h after ethanol treatment provided a less reliable predictor of adult neuron loss. The findings show that ethanol-induced neonatal apoptosis often causes immediate neuron deficits that persist in adulthood, and furthermore suggests that the brain may have limited capacity to compensate for ethanol-induced neuron loss.

## Introduction

In neonatal brain development there is a period of apoptotic neuron loss, or “normal programmed cell death” that eliminates a large fraction of neurons. This pruning of excess neurons is thought to be key developmental mechanism that determines adult neuron number. In mice, the peak episode of this apoptotic event occurs mainly between embryonic day 17 and postnatal day 7 (PD7), depending on the brain region (Forger et al., 2004; Ahern et al., 2013; Mosley et al., 2017). At approximately the same developmental period, ethanol exposure can cause a dramatic spike of apoptosis in some brain areas. Like normal programmed cell death, the brain areas affected by ethanol vary depending on the developmental age. Exposure around birth preferentially affects ventral hypothalamic and ventral thalamic regions whereas exposure from P0-P7 affects dorsal thalamic, hippocampal, and striatal regions, and the cerebral cortex is most affected at around P7 (Ikonomidou et al., 2000). Like normal programmed cell death, ethanol-induced apoptosis reduces adult neuron number, as shown by estimates of total neuron number in several brain regions of adult animals that were treated with neonatal ethanol (Bonthius and West, 1991; Napper and West, 1995a; Goodlett and Eilers, 1997; Livy et al., 2003; Tran and Kelly, 2003; Dursun et al., 2013; Gursky et al., 2019; Smiley et al., 2021).

There is interest in developing treatments to prevent or reverse the effects of neonatal ethanol, and thus it is of interest to know specifically which brain regions and cell types are most vulnerable. However, our current understanding of this issue is patchy. Estimates of neuron loss in adult animals have been done in a limited number of regions, and differences in dose and timing of ethanol treatment make it difficult to compare studies. A more common approach has been to estimate the number of apoptotic cells, using histological sampling within hours or days after ethanol treatment. However, interpretation is complicated by the short duration of the apoptotic response, and differential timing of the response between regions. As a result, substantial apoptotic events that occur a few hours before or after the time of sampling may be overlooked (Olney et al., 2002a; Tenkova et al., 2003). Additionally, it has been suggested that the brain may have some capacity to overcome apoptosis after neonatal lesions, perhaps by downregulating ongoing normal programmed cell death or replacing neurons by neurogenesis or neuronal migration (Mooney and Napper, 2005; Fagel et al., 2006; Smiley et al., 2019). However, it is notable that there is little direct evidence that these compensatory mechanisms occur in significant magnitude to overcome large neuron deficits (Salmaso et al., 2014; Shin et al., 2020).

As an alternative method to evaluate ethanol-induced apoptosis in the neonatal brain, the current study used stereological methods to estimate the cumulative neuron loss that occurred by 8 h after P7 ethanol, or by early adulthood (P70). These results were then compared with a more traditional approach that estimated apoptotic cell number at 8 h after ethanol treatment. Neuron number estimates were taken from several brain regions to obtain an initial evaluation of the regional selectivity of the ethanol effect. Regions were selected because they were previously shown to have robust apoptotic response to P7 ethanol and/or they are representative of distinct forebrain circuitries.

## Materials and methods

A total of 40 C57BL/6J mice were used for this study. Mice originated from 6 different mouse litters with 5–8 animals per litter. Each litter was divided into ethanol and saline treatment groups matched as closely as possible for sex and body weight. At P7, pups were subcutaneously injected with saline or 2.5 g/kg ethanol per injections, with 2 injections separated by 2 h, as previously described (Ikonomidou et al., 2000; Saito et al., 2007). Three of the litters (*N* = 8 saline and 9 ethanol mice) were transcardially perfused 8 h after the first injection, with 4% buffered paraformaldehyde containing 4% sucrose. The remaining litters (*N* = 11 saline and 12 ethanol mice) were weaned at P25-30 and perfused at P70 with 4% buffered paraformaldehyde. The P70 mice used in this study are a subset of the 5 litters used in a previous study (Smiley et al., 2021). The 3 litters at P70 were selected so that average body weight (3.6 +/−0.4 g) was matched as closely as possible to that of the P7 litters (3.3 +/− 0.1 g). All brains were postfixed for 2–4 days and embedded in agar blocks so that matched ethanol and saline treated brains from each litter were simultaneously sectioned and processed (Nagamoto-Combs et al., 2016; Smiley et al., 2019). Blocks were coronally sectioned at 50uM thickness, and a series of every 2nd or 3rd (P7) or 3rd or 4th (P70) consecutive section was dried on chrom-alum subbed slides before staining with thionin and coverslipping with permount. For cleaved caspase-3 (CC3) immunolabeling, free-floating sections were exposed to 1:400 dilution of primary antibody (Cell signaling catalog #9664) that was visualized using the streptavidin-biotin-peroxidase method with diaminobenzidine as the reaction product, and then mounted and dried on subbed slides before coverslipping with permount. All procedures were approved by the Nathan Kline Institute IACUC and were in accordance with NIH guidelines for the proper treatment of animals.

Stereological estimates of total Nissl-stained neuron numbers used the Nv x Vref method (Gundersen et al., 1988). Cell sampling was done using ImageJ software to control a motorized Nikon E-600 microscope equipped with Ludl X, Y, and Z motors, a Heidenhain z-axis microcator, and a Basler acA5472-17 camera. The full rostral-caudal extent of each area was sampled in evenly spaced sections through each structure, using an average of 7.1 +/− 2.4 sections per neuron number estimate (mean +/− S.D., *N* = 353 estimates of total neuron number). In each section, the outlined area of interest was sampled with an evenly spaced grid of sites. At each site a z-stack of six images with 1- or 1.5-micron z-spacing was saved to a computer, and an optical disector counting box was then drawn on each z-stack using the first slice as an upper guard zone, the next 3 slices as a counting box, and the remaining slices as a bottom guard zone. All z-stacks were taken using a 1.3 N.A. 100x objective. Neurons were distinguished from glia by morphological features including their well stained cytoplasm and prominent nucleolus. Separate counts of apoptotic nuclei used the same z-stacks as for total cell number but with a larger counting box. Apoptotic nuclei were easily identified by darkly stained balls of dense chromatin, consistent with previous descriptions (Olney et al., 2002a).

Across all estimates of total neuron number, the average number of disectors (157 +/49) and number of cells counted (190 +/−84) was adjusted to obtain adequate precision, and in most cases the coefficient of error (CE) (Dorph-Petersen et al., 2009) was <0.1. Exceptions were the anterior dorsal thalamic nucleus in ethanol treated animals (average CE = 0.12), that had severely reduced cell numbers in some brains, and the anterior medial thalamic nucleus (average CE = 0.12 in both saline and control animals) that had inhomogeneous cell density across its rostral-caudal extent that resulted in high CE’s. Additionally, CE’s for estimates of pyknotic cell numbers in ethanol treated animals had CE’s ranging from 0.11 to 0.16, due to low number of apoptotic cells in some brains. In saline-treated animals, pyknotic cell numbers were much lower, and are presented here only to confirm that ethanol-induced apoptosis is much higher than background. On-slide section thickness, that was measured to calculate z-axis tissue shrinkage, did not differ significantly between saline-and ethanol-treated animals in any experiment, but there was a difference between ages. Comparing the average thickness from each of 5 areas that were counted at both ages, the thickness at P7 (10.0 +/− 1.1 microns) was less than that at P70 (11.6 +/−0.3 microns, *t*(8) = −3.0, *p* < 0.02, independent samples *t*-test).

**Figure 1:**
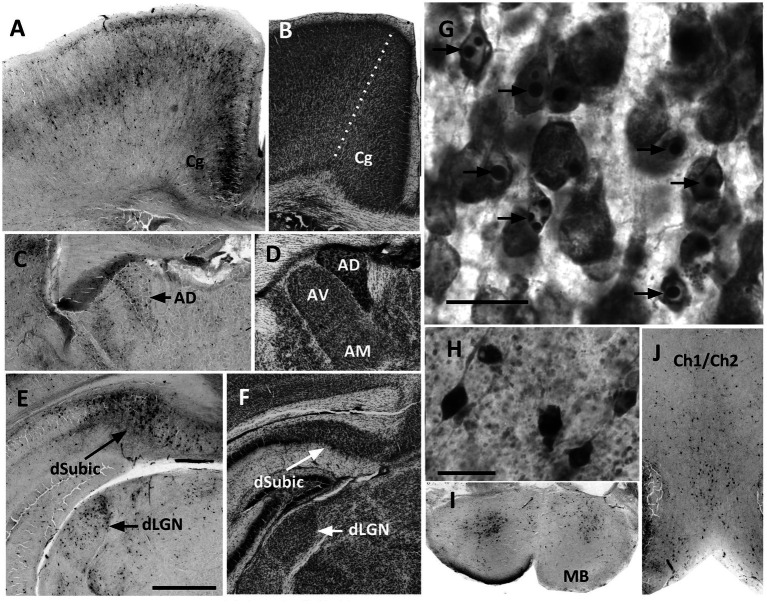
In P7 brains, identification of apoptotic neurons by either CC3-immunolabeling or Nissl staining confirmed an obvious apoptotic response at 8 h after the initiation of treatment. **(A)** CC3-immunolabeling showed a high density of apoptotic cells in the cingulate cortex (Cg), that was present in all cortical layers but were especially dense in layer II and III/IV. **(B)** A Nissl-stained section shows the border (dotted line) of cingulate cortex with motor cortex laterally. Cingulate cortex was distinguished by the dense cell packing in layer II. **(C)** CC3 cell density was consistently high in the anterior dorsal nucleus of the thalamus (AD). **(D)** In Nissl-stained sections the AD nucleus has a characteristic dense appearance. The anterior ventral (AV) and anterior medial (AM) nuclei are also easily identified, although at P7 the border separating the two was somewhat indistinct. **(E)** The dorsal subiculum (dSubic) and dorsal lateral geniculate nucleus (dLGN) had a high density of CC3 cells after P7 ethanol. **(F)** In Nissl-stained sections the dSubic and dLGN were easily identified. **(G)** Higher magnification image of Nissl-stained cortex shows the typical appearance of apoptotic cells, which were easily identified by characteristic darkly stained ovals of condensed chromatin within the nucleus. **(H)** Higher magnification shows the appearance of CC3-immunolabeled cells, most of which had the appearance of neurons. **(I,J)** CC3-immunolabeling was prominent in the cell scattered throughout the mammillary bodies (MB) and the region of the medial septal nucleus/vertical diagonal band (Ch1/Ch2). Scale bar in *E* = 0.5 mm, and applies to all images except **(G,H)**. Scale bars in **(G,H)** = 20μM.

Brain structures were identified as in the Allen Institute Brain Atlas (AIBA, 2011) unless otherwise stated. The anterior dorsal, anterior ventral, anterior medial, and dorsal lateral geniculate (dLGN) thalamic nuclei usually had unambiguous borders. However, the boundary separating the anterior ventral from anterior medial nucleus was less clear in P7 brains, and for this reason these nuclei were only measured in P70 brains. The mammillary body was easily identified, and cell counts included both the medial and lateral mammillary nuclei. The mammillary bodies were excluded from analysis of P7 brains due to tight cell packing in some brains that hindered cell counting. The borders of the medial septum/vertical diagonal band nuclei were identified as in our previous publication (Smiley et al., 2021), and to be consistent with that study we refer to this region as the Ch1/Ch2 nucleus, indicating that it is coextensive with the distribution of cholinergic neurons in this region (Mesulam et al., 1983). Briefly, this region is defined by the large Nissl-stained neurons in the medial septal area and vertical limb of the diagonal band, extending caudally to rostral tip of the third cerebral ventricle at the ventral surface of the brain, where there is often a separation between the vertical diagonal band and the horizontal diagonal band. The boundaries of whole neocortex were defined as in our previous studies (Smiley et al., 2015, 2019). Briefly this region includes most of the medial and lateral cortical surface, but excludes the lateral dysgranular limbic areas (i.e., pyriform, insular, and entorhinal cortex) and excludes retrosplenial cortex in sections caudal to the corpus callosum. Separate analyses of cingulate cortex used boundaries that corresponded approximately to areas 24a/24b of Vogt and Paxinos (2014). However, for simplicity we defined the rostral boundary at the rostral tip of the corpus collosum, and the caudal boundary at the rostral tip of the dorsal hippocampus. Dorsally, the border with motor cortex was identified by the comparatively high cell density in layer II of cingulate cortex (Figure 1B). The dorsal subiculum was identified as in the Allen Institute Mouse Brain Atlas (AIBA, 2011) thus including the subiculum in all sections where its lateral border abutted the hippocampal CA1 region, but excluding sections where its lateral border merged with the ventral subiculum. Thus, this boundary is defined by the shape of the subiculum as it wraps around the hippocampus CA1 region. While its volume was reasonably consistent in P70 brains, it was less reliable in P7 brains where the curvature of the hippocampus was still immature, and for this reason the dorsal subiculum was only measured in P70 brains.

### Statistical analysis

Mice were taken from 3 litters at each age (P7 and P70). Within each litter, animals were selected so that ethanol and saline treated animals had approximately equal distributions of sex and body weight. Estimates of cell number were analyzed using two-way analysis of variance (ANOVA) models with treatment group, age, and sex included as independent variables, and litter as a blocking factor. Main effects of sex or interactions with sex were not encountered in any analyses. Statistical analysis was conducted using IBM SPSS version 24 software. We used mean ± S.D. and percent differences [i.e., (1-ethanol/saline)*100%] to describe the sample and quantify associations.

## Results

Identification of apoptotic neurons by either CC3-immunolabeling or Nissl staining confirmed that P7 ethanol injections caused an obvious apoptotic response at 8 h after the initiation of treatment (Figure 1). Qualitatively, the distribution of apoptotic cells was similar in Nissl-stained sections and CC3-immunolabeled sections. As previously described, the response to P7 ethanol was dense in the anterior thalamic nuclei, and in several regions closely associated with these nuclei including the dorsal subiculum, mammillary bodies, and the cingulate, prefrontal and retrosplenial regions of midline cerebral cortex. Additionally, a comparatively dense apoptotic response was present in regions that do not have strong direct connections with the anterior thalamus, including the dLGN, Ch1/Ch2 region of the basal forebrain, and most of the dorsal lateral surface of the cerebral cortex.

### Ethanol-induced neuron deficits are comparable at P7 and P70

**Figure 2:**
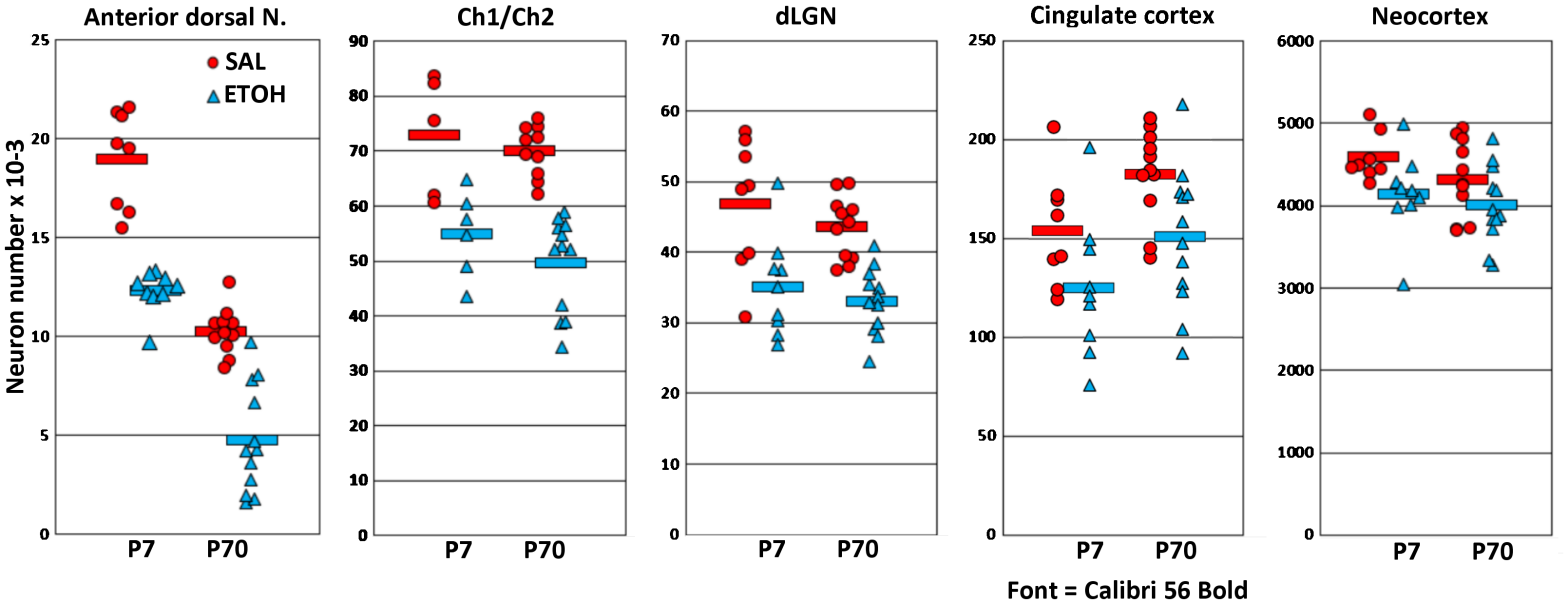
Graphs compare estimates of neuron number at P7 and P70 in 5 different areas, in saline (SAL) and ethanol (ETOH) treated mice. The graphs are ordered from left to right by severity of the ethanol effect. In all regions, the reduction of neuron number was not significantly different at P7 compared to P70. Ethanol caused the largest reduction in the anterior dorsal nucleus, and this nucleus also had a striking age effect, such that neuron number was higher at P7 than P70 in both the saline and ethanol treatment groups.

Stereological estimates of total neuron number in Nissl-stained sections were used to compare neuron loss already present at 8 h after ethanol treatment with neuron loss in P70 animals. Several brain regions were selected based on their robust apoptotic response, and because they were representative of distinctly different functional systems in the forebrain. As described below, each of these regions had significant neuron loss at P7 that was not significantly different from the neuron loss at P70 (Figure 2).

In the anterior dorsal thalamic nucleus, ANOVA showed a main effect of ethanol treatment [*F*(1,31.6) = 125, *p* < 0.001] and a main effect of age [*F*(1,4.0) = 42.4, *p* < 0.001], but there was not a significant interaction of treatment and age [*F*(1,30) = 1.9, *p* < 0.18]. The main effect of age was due to a normal developmental neuron loss that caused nearly 50% reduction of neuron number from P7 to P70. Notably, this age-dependent neuron loss was also present in ethanol-treated animals, even after a large ethanol-induced neuron deficit at P7. The main effect of ethanol treatment was due to an average reduction of total cell number that was similar at P7 (average decrease of 6,686 neurons) or P70 (5,507 neurons). Because of the age-related decrease in total neuron number, these changes correspond to 35% of total neurons at P7, or 54% at P70. Estimates of total neuron number in additional untreated control animals confirmed that total neuron number consistently decreases after P7 in this nucleus (data not shown).

In the Ch1/Ch2 nuclei of the basal forebrain, only 5 saline-control and 6 ethanol-treated animals were available at P7 because this region was damaged during processing in one litter. In this region there was a main effect of ethanol treatment [*F*(1,24.6) = 52.3, *p* < 0.001]. There was not a main effect of age [*F*(1,3.2) = 1.3, *p* = 0.36], nor was there a significant interaction of treatment and age [*F*(1,30) = 1.9, *p* < 0.18]. Thus, the average 24% reduction of total neurons at P7 was not significantly different from the 29% reduction at P70.

In the dLGN there was a main effect of ethanol treatment [*F*(1,31.7) = 43.5, *p* < 0.001]. There was not a main effect of age [*F*(1,4.0) = 0.48, *p* = 0.54], nor was there a significant interaction of treatment and age [*F*(1,30) = 0.24, *p* < 0.63]. Thus, the average 25% reduction of total neurons at P7 was not significantly different from the 24% reduction at P70.

In the cingulate cortex, there was a main effect of ethanol treatment [*F*(1,30.7) = 10.2, *p* < 0.01]. There was not a main effect of age [*F*(1,4.0) = 3.8, *p* = 0.12] nor was there an interaction of treatment and age [*F*(1,30) = 0.07, *p* < 0.78]. In this region ethanol caused reductions of about 19% at P7 and 18% at P70.

In total neocortex, there was a main effect of ethanol treatment [*F*(1,30.7) = 7.9, *p* < 0.01]. There was not a main effect of age [*F*(1,4.0) = 1.0, *p* = 0.37] nor was there a significant interaction of treatment and age [*F*(1,30) = 0.71, *p* < 0.41]. In this region ethanol caused reductions of about 10% at P7 and 7% at P70.

In addition to the above age comparisons, we estimated neuron number only at P70 in other nuclei functionally associated with the anterior dorsal thalamic nuclei, including the anterior ventral and medial thalamic nuclei, the dorsal subiculum, and the mammillary bodies. As expected from the distribution of apoptotic neurons at P7, there was robust neuron loss in the anterior ventral 36% [*F*(1,17) = 28.3, *p* < 0.001] anterior medial 36% [*F*(1,17) = 64.8, *p* < 0.001] and dorsal subiculum 31% [*F*(1,17) = 20.8, *p* < 0.001]. Neuron loss was less pronounced in the mammillary bodies 15% [*F*(1,17) = 7.9, *p* < 0.05].

**Figure 3:**
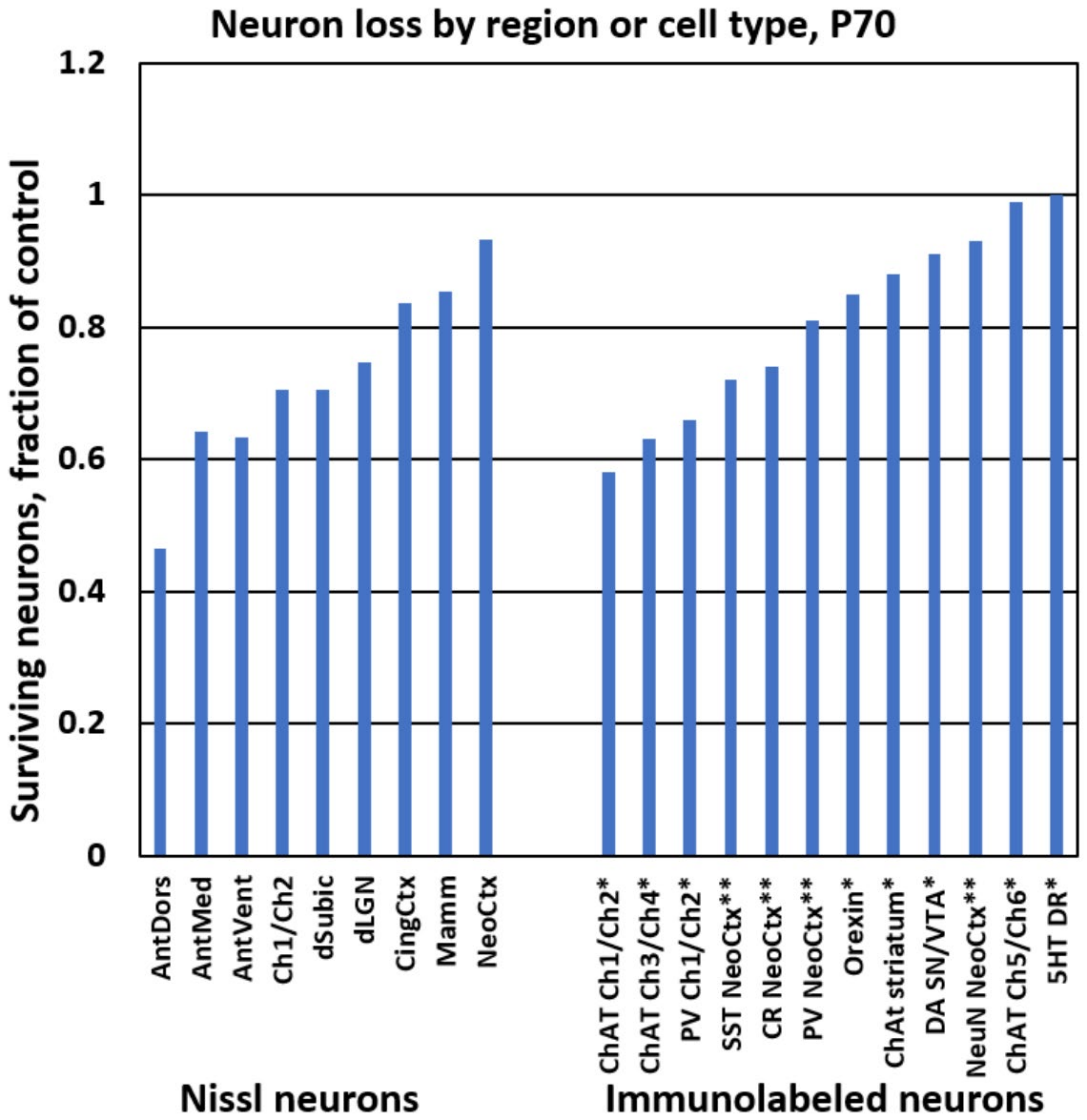
Bar graphs show the ethanol-induced neuron loss at P70 from different brain regions in the present study (“Nissl neurons,” at left). For comparison, at right are stereological estimates of immunolabeled neuron populations from previous studies using the same treatment paradigm. * Results are from Smiley et al. (2021). ** Results are from Smiley et al. (2019). Abbreviations: 5HT DR, serotonin neurons of dorsal raphe; AntDors, anterior dorsal thalamic nucleus; AntMed, anterior medial thalamic nucleus; AntVent, anterior ventral thalamic nucleus; ChAT, choline acetyl transferase immunolabeled cells; Ch1/Ch2, medial septum and vertical diagonal band region; Ch3/Ch4, horizontal diagonal band and nucleus basalis region; Ch5/Ch6, pedunculopontine and lateral dorsal tegmental nuclei; CingCtx, cingulate cortex; CR, calretinin immunolabeled cells; DA SN/VTA, dopamine cells of substantia nigra and ventral tegmental area; dLGN, dorsal lateral geniculate nucleus; dSubic, dorsal subiculum; Mamm, mamillary bodies; NeoCtx, neocortex; NeuN, neuronal nuclear antibody immunolabeled cells; Orexin, orexin cells of the hypothalamus; PV, parvalbumin immunolabeled cells; SST, somatostatin immunolabeled cells.

The above estimates show that some regions are more affected by P7 ethanol. To provide context for these findings, Figure 3 displays the number of surviving neurons as a fraction of control in different regions from the present study, and also in several immunolabeled neuron populations that were evaluated in our previous studies, using the same ethanol treatment paradigm and cell counting methods (Smiley et al., 2019, 2021). Currently, it remains unclear whether reduction of immunolabeled cells corresponds completely to neuron loss or might be partially caused by decreased immunolabeling. Nevertheless, these comparisons suggest that especially large reductions such as those in the anterior thalamus and in immunolabeled cholinergic and GABAergic neurons provide useful biomarkers for the effects of neonatal ethanol.

### Estimates of the number of apoptotic nuclei are poor predictors of total neuron loss

**Figure 4:**
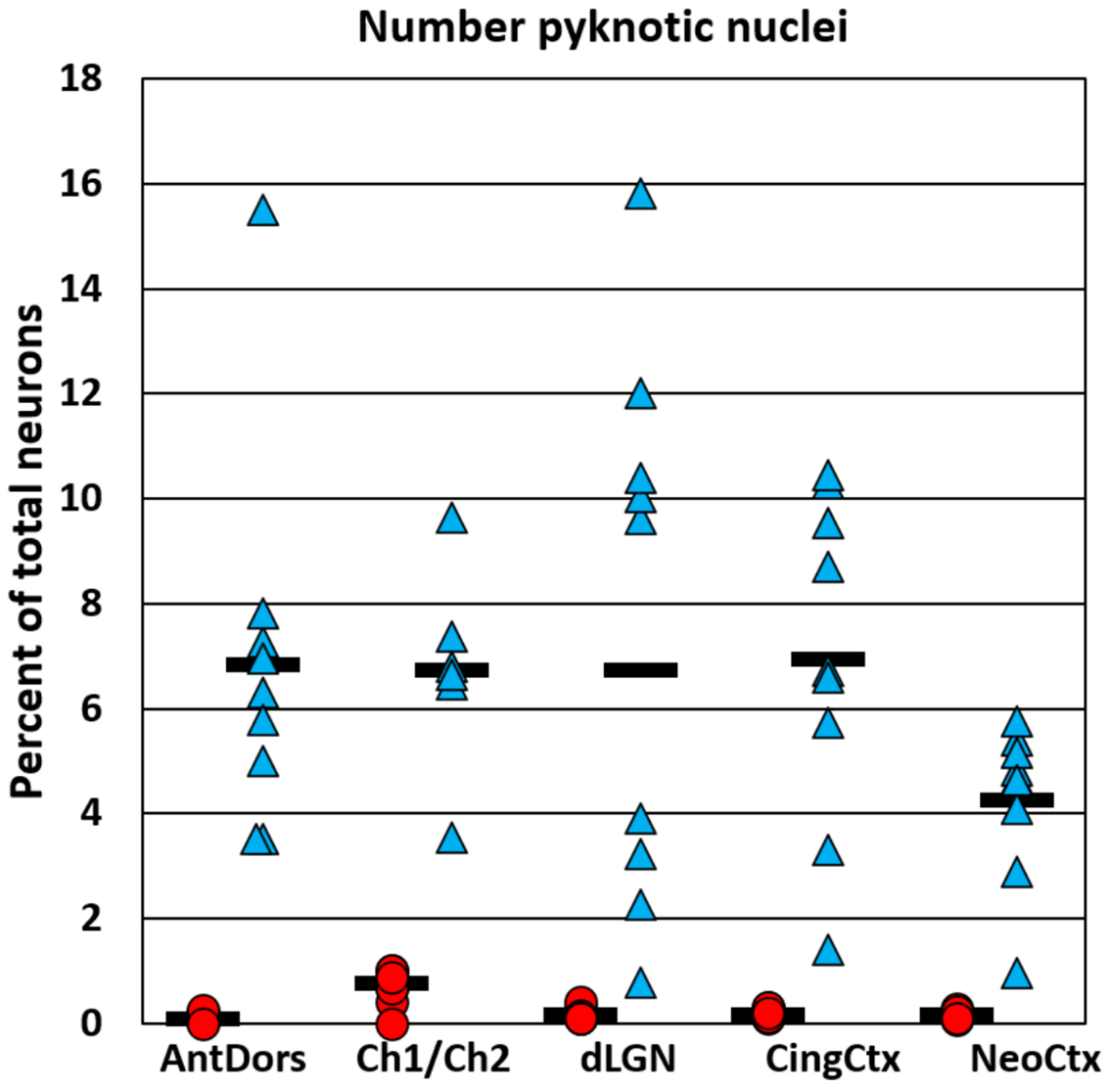
Graphs show the number of Nissl-stained apoptotic cells in each region, expressed as a percent of the total neuron number. The number of apoptotic cells was estimated in P7 brains at 8 h after the onset of ethanol treatment. In each region ethanol treatment caused a robust increase in the number of apoptotic cells compared to saline controls. However, the percent of total cells identified as apoptotic was only a fraction of the total cumulative neuron loss (see Figure 2), indicating that most neurons that died in response to ethanol have already been cleared and are no longer detectable by this method.

Besides estimating total neuron loss, we additionally estimated the number of Nissl-stained apoptotic nuclei in P7 animals, using, using the same image stacks that were collected to estimate total cell number. While many apoptotic cells had the appearance of neurons, this estimate likely also includes some fraction of non-neuronal cells. In saline controls, the number of apoptotic cells, expressed as a percent of the total neuron number, did not exceed 0.8% in any region. In ethanol-treated animals, the average number of apoptotic cells was 4.2% of the total neuron number in neocortex, and between 6.7 and 7.6% in the other regions examined (Figure 4). Thus, in all regions the number of visible apoptotic cells was only a fraction of the cumulative neuron loss at 8 h after ethanol treatment (ranging from 20% in the anterior dorsal nucleus to 43% in neocortex). Additionally, there was not a significant correlation between the number of apoptotic neurons and the total neuron loss across the five regions examined at P7 [r(8) = −0.66, *p* = 0.23]. Thus, the number of apoptotic nuclei sampled at 8 h after treatment is a poor predictor of total neuron loss.

## Discussion

Fetal alcohol can have distinct effects on brain development depending on the timing and dosage of ethanol exposure. In the present study we employed a specific exposure paradigm that is optimized to cause a spike of cellular apoptosis across several regions of the forebrain. While this apoptotic response has been extensively described, it is less well known whether the apoptotic response consistently causes permanent neuron loss. It has been suggested that the brain might have some capacity to compensate for the initial apoptotic loss of neurons by altering the course of still ongoing normal programmed cell death, or enhancing neurogenesis, and that some neuron populations may persist with altered or down-regulated phenotypes (e.g., Smiley et al., 2019). The results of the present study do not exclude that these compensatory processes occur to some extent in some neuron populations. However, they show in several brain regions that the magnitude of ethanol-induced neuron reduction at 8 h after ethanol exposure is comparable to the reduction found when animals are allowed to grow to adulthood. Thus, a common result of ethanol-induced apoptosis at the neonatal age is an immediate and lasting reduction of neuron number.

Previous studies of the P7 ethanol treatment used in the present study concluded that the mechanism of cell death is mainly or completely apoptotic, without obvious excitotoxic cell death (Olney et al., 2002b). Most studies that characterized the severity of this response have quantified the number or density of apoptotic cells, visualized with by methods including Nissl staining, CC3 immunolabeling, fluorojade labeling, TUNEL labeling, or deOlmos silver staining. A limitation of these methods is that the sampling is limited to the time of death and may overlook apoptosis that occurs earlier or later (Olney et al., 2002a; Young and Olney, 2006). Direct comparisons noted that CC3 immunolabeling peaks comparatively early (at approximately 2–10 h depending on the region) whereas Nissl-stained pyknotic nuclei are prominent somewhat later than CC3, and deOlmos silver staining reaches its maximum at about 12–20 h (Ikonomidou et al., 2000; Olney et al., 2002a,b; Tenkova et al., 2003). Olney and colleagues concluded that deOlmos staining after 12 h gives a more complete picture of the full extent of neuron loss. Using the deOlmos method, and the same ethanol-treatment paradigm as the present study but in rats, they estimated that approximately 20% of neurons were apoptotic in cingulate cortex, subiculum, and lateral dorsal thalamic nucleus (Ikonomidou et al., 2000). In the present study of mouse brains, we used an alternative approach that quantifies total neuron number, and thus provides an estimate of cumulative neuron loss that has occurred up to the time of sampling. Using regions comparable to the ones used by Ikonomidou et al. (2000), this method showed neuron losses in the range of 15–30%, thus approximately consistent with estimates derived from deOlmos silver staining. Based on these comparisons, we suggest that stereological quantification Nissl-stained neuron number provides a practical method to quantify regional differences and the developmental timing of ethanol-induced or normal developmental neuron loss.

We additionally quantified the number of apoptotic nuclei at 8 h after P7 ethanol and found that their total number was only a fraction of the cumulative neuron loss. This indicates that many apoptotic nuclei are already fully cleared after 8 h, consistent with previous estimates that CC3 immunolabeling in apoptotic cells is only present for 2–3 h (Olney et al., 2002a). Thus, while these markers of apoptotic cells are useful to confirm the presence of apoptotic response, they are less reliable predictors of regional differences or total neuron loss.

Recently, we found that the Ch1/Ch2 nuclei of the basal forebrain have substantial neuron loss after P7 ethanol (Smiley et al., 2021) and a goal of the present study was to compare the loss in Ch1/Ch2 with that of other brain regions that have pronounced apoptotic response to P7 ethanol. Comparisons across areas showed that the anterior dorsal nucleus has especially severe neuron loss of about 50%, as expected from a previous report of volume loss in this nucleus (Wozniak et al., 2004). Somewhat less severe neuron reductions were found in brain regions that have strong synaptic and/or functional relationship with the anterior dorsal nucleus, including the anterior ventral and anterior medial nuclei, the mammillary bodies, the dorsal subiculum, as well as midline cingulate cortex that receives major thalamic inputs from the anterior thalamic nuclei. These regions are components of the hippocampal-anterior thalamic circuitry that is strongly involved in spatial memory and navigation (Aggleton et al., 2010; Jankowski et al., 2013), and these functions are notably disrupted by P7 ethanol treatment (Marquardt and Brigman, 2016). In the Ch1/Ch2 nuclei the magnitude of neuron loss was comparable to or slightly less than that in the anterior thalamic nuclei. Ch1/Ch2 has only minor connections with anterior thalamic region (Agostinelli et al., 2019), but it does project to hippocampal regions including the subiculum and CA1 region that are likely to also have significant cell loss (Ikonomidou et al., 2000). At present, it is not clear why certain brain areas are especially vulnerable to P7 ethanol. It is possible that they have a synchronous schedule of neuron development, and/or they share a common pharmacological profile.

The present study focused on several brain regions known to have a strong apoptotic response to P7 ethanol and provides only a partial listing of brain areas that are likely to have substantial neuron loss. Other areas with high densities of apoptotic neurons after P7 ethanol include the thalamic reticular nucleus, thalamic lateral dorsal nucleus, superior colliculus, CA1 region of hippocampus, as well as other cortical areas in the mid-sagital sulcus (Ikonomidou et al., 2000; Tenkova et al., 2003) and prominent cell loss was shown in the thalamic nucleus reuniens (Ikonomidou et al., 2001; Gursky et al., 2019). Extensive neuron loss was also shown in cerebellar cortex and associated inferior olivary and deep cerebellar nuclei (Napper and West, 1995b; Pierce et al., 1999; Green et al., 2002; Nirgudkar et al., 2016). In cerebellar cortex, Purkinje cells were reduced by as much as 25–50% depending on the dose and timing of ethanol exposure, and the region of cortex. Although the peak vulnerability in the cerebellum was described as occurring around P4-5 in rats, there was still substantial cell loss at P6-7 (Hamre and West, 1993). While neuron deficits are likely in the above-mentioned areas, it is important to note that some neuron populations are comparatively unaffected by P7 ethanol. Using the same treatment as the current study, we previously found only minor changes in several brainstem nuclei (Figure 4), and in the present study we replicated our previous finding that whole neocortex has comparatively small deficits in total neuron number (Smiley et al., 2015, 2019).

In the present study we found in control brains that total neuron number in the anterior dorsal nucleus is not reduced to adult levels until after P7. To our knowledge, the timing of normal programmed cell death in the anterior thalamic nuclei has not been systematically studied. In most other forebrain regions, the main episode of neonatal programmed cell death is thought to be mainly completed by around P7 (Ahern et al., 2013; Mosley et al., 2017). However, there is precedence for later normal programmed cell death in some regions. For example, the substantia nigra has an early peak of apoptosis around P3 and a later peak around P14 (Oo and Burke, 1997). In the dLGN, total neuron number was shown to continue decreasing after P10, but those estimates were done in albino mice (Heumann and Rabinowicz, 1980), whereas a study of the dLGN in rats concluded that the peak of normal developmental apoptosis occurs around P1 and is complete by P7 (Zacharaki et al., 2010).

In summary, our findings show that estimation of total neuron number within hours of P7 ethanol provides a reliable method to assess regional differences in neuron loss caused by neonatal ethanol. Results obtained with this approach showed that a common outcome of ethanol-induced apoptosis is immediate and lasting neuron loss. Comparison between regions showed that neuron loss was most pronounced in the anterior dorsal thalamic nucleus, but other neuron populations in the anterior thalamus, subiculum and Ch1/Ch2 were also strongly affected, whereas comparatively modest neuron loss was detected in neocortex.

## Data availability statement

The raw data supporting the conclusions of this article will be made available by the authors, without undue reservation.

## Ethics statement

The animal study was reviewed and approved by Nathan Kline Institute IACUC.

## Author contributions

JS involved in the study design and conception, data acquisition and analysis, and writing the manuscript. CB, BM, PV, and SC-B engaged in data acquisition and analyses and commenting and editing the drafts. MS and DW engaged in the study design and conception and commenting and editing the drafts. All authors contributed to the article and approved the submitted version.

## Funding

This work was supported by R01AA023181 to MS, DW, and JS.

## Conflict of interest

The authors declare that the research was conducted in the absence of any commercial or financial relationships that could be construed as a potential conflict of interest.

## Publisher’s note

All claims expressed in this article are solely those of the authors and do not necessarily represent those of their affiliated organizations, or those of the publisher, the editors and the reviewers. Any product that may be evaluated in this article, or claim that may be made by its manufacturer, is not guaranteed or endorsed by the publisher.
